# Novel microscope-based visual display and nasopharyngeal registration for auditory brainstem implantation: a feasibility study in an ex vivo model

**DOI:** 10.1007/s11548-021-02514-x

**Published:** 2021-11-18

**Authors:** Milovan Regodić, Christian F. Freyschlag, Johannes Kerschbaumer, Malik Galijašević, Romed Hörmann, Wolfgang Freysinger

**Affiliations:** 1 Department of Otorhinolaryngology, Medical University of Innsbruck, Innsbruck, Austria; 2 Department of Radiation Oncology, Medical University of Vienna, Vienna, Austria; 3 Department of Neurosurgery, Medical University of Innsbruck, Innsbruck, Austria; 4 Department of Neuroradiology, Medical University of Innsbruck, Innsbruck, Austria; 5 Neuroimaging Research Core Facility, Medical University of Innsbruck, Innsbruck, Austria; 6 Department of Anatomy, Histology and Embryology, Medical University of Vienna, Vienna, Austria

**Keywords:** Auditory brainstem implant, Visual guidance, Electromagnetic tracking, Registration, Neuronavigation

## Abstract

**Purpose:**

An auditory brainstem implant (ABI) represents an alternative for patients with profound hearing loss who are constrained from receiving a cochlear implant. The positioning of the ABI electrode influences the patient’s auditory capacity and, therefore, quality of life and is challenging even with available intraoperative electrophysiological monitoring. This work aims to provide and assess the feasibility of visual-spatial assistance for ABI positioning.

**Methods:**

The pose of the forceps instrument that grasps the electrode was electromagnetically navigated and interactively projected in the eyepieces of a surgical microscope with respect to a target point. Intraoperative navigation was established with an experimental technique for automated nasopharyngeal patient registration. Two ABI procedures were completed in a human specimen head.

**Results:**

An intraoperative usability study demonstrated lower localization error when using the proposed visual display versus standard cross-sectional views. The postoperative evaluations of the preclinical study showed that the center of the electrode was misplaced to the planned position by 1.58 mm and 3.16 mm for the left and the right ear procedure, respectively.

**Conclusion:**

The results indicate the potential to enhance intraoperative feedback during ABI positioning with the presented system. Further improvements consider estimating the pose of the electrode itself to allow for better orientation during placement.

**Supplementary Information:**

The online version contains supplementary material available at 10.1007/s11548-021-02514-x.

## Introduction

Restoring speech understanding in deaf patients by stimulating the auditory nerve through a cochlear implant (CI) is a routine procedure [1]; however, inner ear anomalies such as bilateral damage of the auditory nerve may constraint receiving a CI. These patients can benefit from an auditory brainstem implant (ABI) [2]. The auditory brainstem implant electrode pad (electrode array) bypasses the cochlea and the hearing nerve to directly stimulate the auditory network on the cochlear nucleus (CN) in the brainstem. Initially, this surgery was indicated for adult patients diagnosed with a neurofibromatosis type 2 (NF2) [3], but nowadays, ABI is also considered for patients with other cochlear malformations as small or absent cochleae at pediatric patients [4].

The placement of the electrode pad onto the curved surface of CN (with an area typically 6 mm $\times$ 7 mm [5]) poses a significant intraoperative challenge for surgeons. The positioning directly influences the levels of speech recognition and whether there are non-auditory side effects in patients while at the same time there is the risk of brainstem injury [6]. Orientation by anatomical landmarks and intraoperative measured evoked auditory brainstem responses (EABRs) are considered gold standard navigation for electrode placement [7]. Anatomical landmarks in proximity to the target structure serve for identification of the CN surface that is not fully visible during surgery; moreover, orientation may be altered due to previous surgeries around the cerbello-pontine angle (CPA). To spot the active auditory context on the CN, EABRs assist in combination with a placement (stimulation) electrode [7]. In current practice, once detected, the optimal position is memorized by the surgeon and sometimes marked in situ and then targeted with the ABI [8].

This work evaluates a recently proposed visual-spatial display for surgical targeting aimed to assist, quantify and verify ABI placement [9, 10]. The proposed system coupled with an innovative nasopharyngeal registration allows reaching accurately the stored position of the ABI determined with the placement electrode. A prototype was used by two neurosurgeons to assess the feasibility in a preclinical setting with a human head specimen.

## Methods

### Intraoperative tracking

**Fig. 1:**
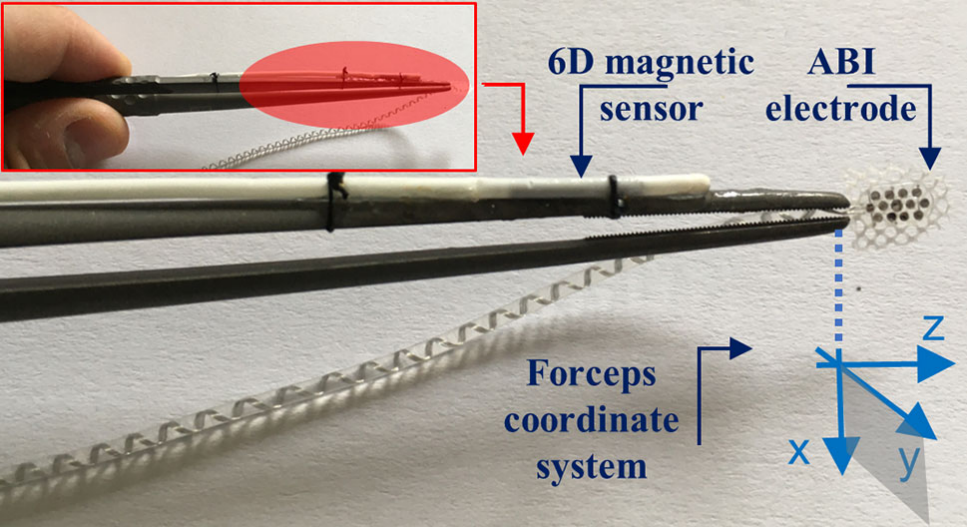
Titanium medical forceps with an in-house mounted 6D magnetic sensor

To enable spatial localization within the 3D space of the patient’s anatomy, a titanium medical forceps (B. Braun AG, Melsungen, Germany), typically used for grasping brainstem electrodes, was navigated using electromagnetic tracking (EMT). A 6D magnetic sensor (0.8 mm x 9 mm, NDI - Northern Digital Inc., ON, Canada) was attached on one tranche of the forceps; the instrument position was pivot-calibrated [11] at the tip. Figure 1 illustrates the setup.

### Patient-to-image registration

**Fig. 2:**
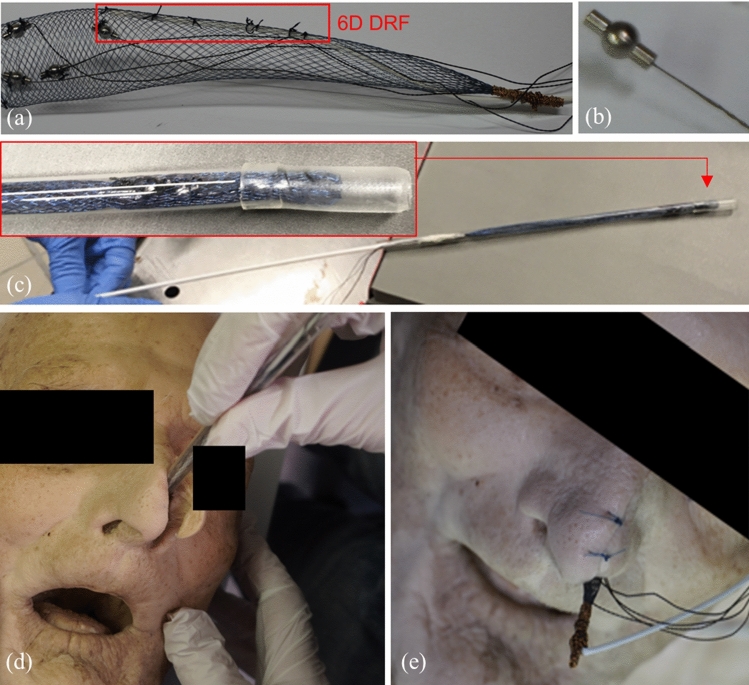
**a** A prototype of the registration device consists of a titanium nasal stent, four titanium spherical fiducials (4 mm $\times$ 8 mm, in-house designed) with isocentrically mounted 5D magnetic sensors (0.5 mm $\times$ 8 mm NDI) acting as fiducials both in image and in patient space and one 6D magnetic sensor (0.8 mm $\times$ 9 mm, NDI) acting as a dynamic reference frame (DRF); **b** a titanium spherical fiducial sample with an isocentrically integrated 5D magnetic sensor; **c** a positioning tube with a pusher ready for the stent assembly deployment inside the nasopharynx; **d** positioning process; **e** final positioning

Preoperative radiological imaging needs to be registered to the intraoperative patient setting to allow intraoperative navigation. An automated nasopharyngeal point-based registration that utilizes titanium spherical fiducials combined with EMT systems serves this purpose. A titanium nasal stent based on the AlaxoStent (Alaxo GmbH, Germany) normally used for breathing enhancement in patients [12]—served to elaborate minimally invasive positioning and stabilization of four spherical fiducials and isocentrically mounted magnetic position sensors in the nasopharynx prior to preoperative imaging (Fig. 2). An algorithm was developed and verified to automatically localize fiducials in preoperative imagery and match these positions with the intraoperative positions provided by integrated magnetic sensors [13].

### Visual display for surgical targeting

The visual display (introduced in [9, 10]) indicates the displacements between the current position of the tracked instrument tip and the designed target point using intuitive, minimalistic, and semi-transparent visual clues superimposed onto the video of the actual surgical site. The three axes of the instrument were encoded with respect to the observer’s viewpoint: the *z*-axis runs longitudinally along the instrument tip while the $x-y$ axes form a plane perpendicular to it, Fig. 1. The *z*-direction represents moving in/out and is termed as “depth”; movements in the $x-y$ plane left/right and up/down and are termed as “lateral.”

The lateral and depth vectorial displacements between instrument-tip and target position were measured and visually represented in a 2D view (e.g., a microscopic view) (Fig., Supp. 1).

The lateral displacement was visualized with a green blob 2D-translated from a red square, the target (Fig., Supp. 1a-d). When the lateral distance is zero, the two visual cues overlap (Fig., Supp. 1d).

The distance in the depth direction is visualized as an interactive circle with a diameter proportional to the depth distance with its origin on the target. In other words, the circle minimizes or maximizes its diameter if the instrument tip is moving toward or away from the target (Fig., Supp. 1e-h). When the depth distance is zero, the circle vanishes (Fig., Supp. 1h). If the instrument is beyond (or overreached) the target, the circle behaves similarly but blinks to signalize for this (Video, Supp. 3, 07:27).

The virtual cues in pixels are scaled to the surgeon’s preferences to provide an intuitive perception of physical dimensions, such as revealing and emphasizing small instrument motions.

When the instrument is out of the defined target range, only the visual cues for target and depth are displayed, and the lateral displacement becomes lateral orientation with a long arrow pointing toward the target (Fig., Supp. 2). Furthermore, the diameter of the depth circle is kept constant.

**Fig. 3:**
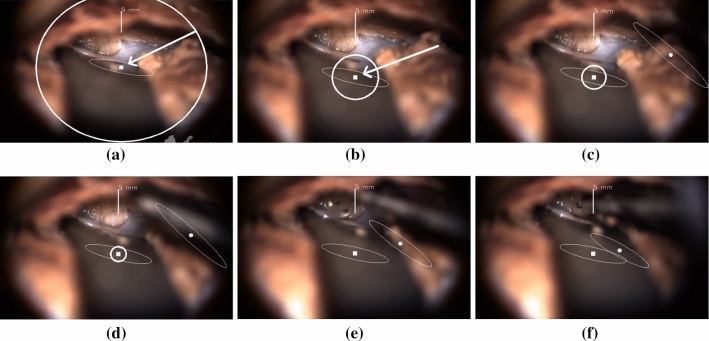
The visual display in the surgical microscope oculars during brainstem electrode placement. The forceps with the electrode is moved from **a** the outside of the defined region toward **f** the target point

An example of the visual display is shown in Fig. 3.

**Fig. 4:**
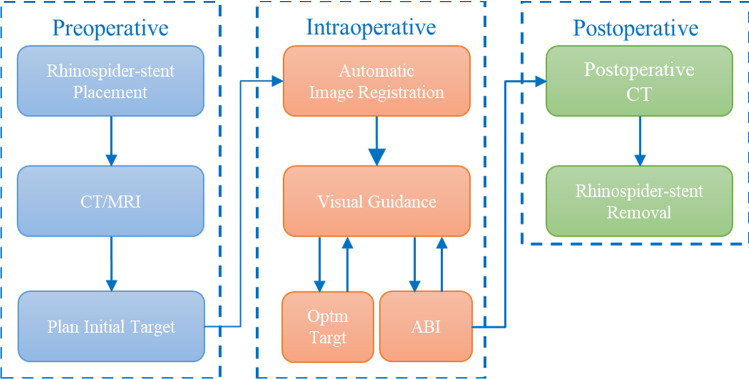
The experiment workflow. “Optm Targt”: the first step to optimize an initially planned target in image and “ABI”: the second step to place the implant on the determined optimal position

### Positional uncertainty

Navigation is perturbated by random noise and measurement errors [14, 15]. Therefore, intraoperative awareness of the accuracy range (“reliability”) is beneficial and could help avoid tissue damage and optimize electrode placement by avoiding directions with higher uncertainty when using the surgical instrument. We modeled these errors as the distribution of TRE (target registration error) [16] to quantify the uncertainty of instrument-tip localization at the surgical target point. The model was implemented using a general approach for first-order approximation [17] and was validated across two other similar algorithms [16, 18]. The chi-square distribution with 95% confidence regions are shown as green and red “error” ellipses in the lateral directions around instrument-tip and target (Fig., Supp. 1a-d).

### Preclinical feasibility study

The experiment workflow depicted in Fig. 4 is described below.

#### Surgeons

Two neurosurgeons that regularly carry out the brain and skull-base surgeries with routine use of different neuronavigation systems and experience in ABI surgery were selected to perform auditory brainstem implantations using the system in a preclinical environment.

#### Specimen

The formalin-embalmed human specimen head was provided by Department of Anatomy at the Medical University of Innsbruck, Innsbruck, Austria. No protected health information/ethics committee approval was required for this kind of study. However, we have followed all required internal regulations for scientific work with donated human bodies [19, 20].

**Fig. 5:**
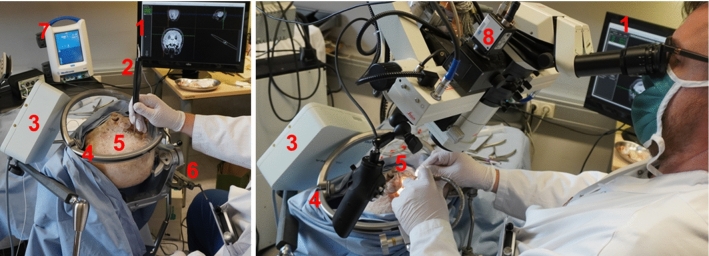
Experimental setup left—estimation of registration quality on a screw and right—a neurosurgeon performing the intervention. In **1**—cross-sectional views screen, **2**—surgical probe, **3**—field generator, **4**—halo ring system with retractors, **5**—surgical site, **6**—Mayfield clamp, **7**—Medtronic Drill System for Neurosurgery, **8**—an industrial camera mounted on the microscope

#### Image registration and surgery planning

Prior to preoperative CT imaging (0.6 mm slice thickness, pixel spacing 0.43 $\times$ 0.43 ${\mathrm{mm}}^2$), the nasal-stent assembly was placed in the nasopharynx and remained there until the end of whole procedures (Fig. 2c–e). A landmark-based registration [21] served to pair positions from magnetic sensors with the centroids of the detected spherical fiducials in images [9, 13]. This process was automated and was repeated multiple times during the intervention.

A preoperative MR scan was also acquired (3T, sequence rapid gradient-echo T1 weighted sequence, slice thickness 1 mm, pixel spacing 0.86 $\times$ 0.86 ${\mathrm{mm}}^2$) without the implanted stent assembly and fused to the preoperative CT scan for surgical planning. The two images were co-registered using customized in-house software [9] that implements an intensity-based method with mutual information and rigid transformation [22]. The initial target point was designed in the foramen of Luschka [23] in fused and blended CT and MR images.

#### Predicted TRE

The predicted TRE at the surgical point of interest in the foramen of Luschka was 1.75 mm and 1.76 mm for the left and the right ear, respectively. The TRE was modeled using [17] with the fiducial configuration obtained from the CT scan; and input FLE (fiducial localization error) [16] resulted as in a combination of 0.1 mm error in image space [13] and 0.7 mm error for EMT in physical space (Aurora V3.1 User Guide, 2018, NDI).

#### Measured TRE

To measure TRE, eight screws (2 mm in diameter and 6 mm in length—Stryker, Kalamazoo, MI, USA) were implanted into the skull around petrous bone and ear regions prior to radiological imaging. However, only 6 were still present and stable at the intervention. Following automated registration, in addition to visually inspecting accuracy on bony anatomical landmarks during surgery, each screw was touched three times with a calibrated pointer (Aurora 6DOF Probe, NDI) and compared pairwise to its detected automatically equivalent in the image [9].

#### Surgical navigation

An Aurora EMT system (NDI) was connected to an in-house implemented image-guided station previously validated on phantoms [9] (Video, Supp. 3).

#### Surgical procedures

Two interventions were completed, on the left and the right ear side of the specimen, in a wet laboratory to closely approximate the intraoperative setting (Fig. 5). Implantation was carried out with close resemblance to the routine implantation in patients. Retrosigmoid craniotomy followed by arachnoidal dissection was performed.

MED-EL, Innsbruck, Austria provided the electrodes (Mi1200 SYNCHRONY PIN ABI and ABI Placement Electrode) [24]. Despite EABRs inaccessibility, target optimization with a placement electrode was followed based only on extrapolated anatomy from the surgeon’s knowledge to adjust the initially designed target position. The system stored the optimal anatomical position by keeping the surgical instrument stable for several seconds at a single location. The renewed target point was updated in the visual interface, enabling accurate guidance to the stored position. After placement in its final position, the ABI electrode was fixed with fibrin glue, and postoperative CT scans were acquired.

#### Assessment of fiducial marker stability

The registration error can be inflated due to the spatial dislocation of four markers due to the procedures, skull drilling, specimen positioning, transportation to the storage and imaging room, etc. It is therefore essential to verify how effective is the nasal stent for marker stabilization. For this purpose, we compared the variability in fiducial position between pre- and postoperatively recorded CT images.

**Table 1:** Experimentally determined TRE for skull-implanted screws with their mean±standard deviation ($\mu \pm \sigma$) for the left and the right ear

	Left ear					Right ear				
	T1	T2	T3	T4	$\mu \pm \sigma$	T5	T6	T7	T8	$\mu \pm \sigma$
Measured (mm)	*NA*	4.0	2.1	1.6	$2.56 \pm 1.27$	1.9	3.1	3.6	*NA*	$2.87 \pm 0.87$
Predicted (mm)	4.0	4.0	3.1	2.4	$3.37 \pm 0.77$	3.1	4.1	4.2	*NA*	$3.8 \pm 0.6$

#### Assessment of usability placement

In parallel to the procedures, the surgeons were instructed to perform dummy electrode positioning on the marked target using the surgical instrument navigated with three system combinations: the presented visual interface (VG) and two other system combinations with cross-sectional views (IGS as a stand-alone and IGS+VG as a multi-modal combination). The measured quantities included localization error represented as the distance between the planned target in the image and the localized position on the electrode after dummy positioning; reaction time and trajectory sum between the start and end time of a localization period.

#### Assessment of final placement

After the procedures, the real electrode position was assessed as the center of the ABI on the surface of the imaged electrode closer to the target point. This position was superimposed and compared to the target position in the primary CT scans. The 3D-vectorial displacement between these two positions was measured as the Euclidian distance, lateral target error and depth target error. The lateral error was calculated as the 2D distance in a plane that lies on the electrode’s surface, whereas the depth error as the 1D distance orthogonal to this plane (Fig., Supp. 4).

## Results

### Evaluation of intrasurgical fiducial variance

The mean deviation in fiducial positions from its base locations was $0.29\,\pm \,0.20$ for the left-side intervention and $1.31\,\pm \,0.30$ for the right-side intervention. A more detailed accuracy inspection for individual fiducials was reported [25].

### Evaluation of image registration

The mean ± standard deviation quantities were measured for the left and the right side intervention. The FRE (fiducial registration error) [16] for six repetitions during usability (dummy) and final positioning gave $0.53\,\pm \,0.1$ mm and $0.44\,\pm \,0.04$ mm. The TRE measured on screws was $2.56 \pm 1.27$ mm and $2.87\,\pm \,0.87$ mm. The numerically predicted TREs on screws using the process described in Sect. 2.5.4 obtained higher values compared to the measured TREs (see Table 1). T2 screw was loosely fixed during evaluation. T1 and T8 were lost.

### Evaluation of usability placement

Table 2 presents the evaluation performed for dummy electrode positioning. IGS and VG contained 4 collected trials each while IGS+VG only 2.

**Table 2:** Experimentally determined mean±standard deviation for localization error as Euclidian distance, trajectory and completion time

System	Euclidean distance	Trajectory	Completion time
	(mm)	(cm)	(s)
IGS	$3.98\pm 1.25$	$84\pm 74$	$65\pm 26$
VG	$2.32\pm 0.61$	$114\pm 54$	$86\pm 19$
IGS+VG	$3.06\pm 0.88$	$34\pm 5$	$42\pm 11$

### Evaluation of final placement

**Fig. 6:**
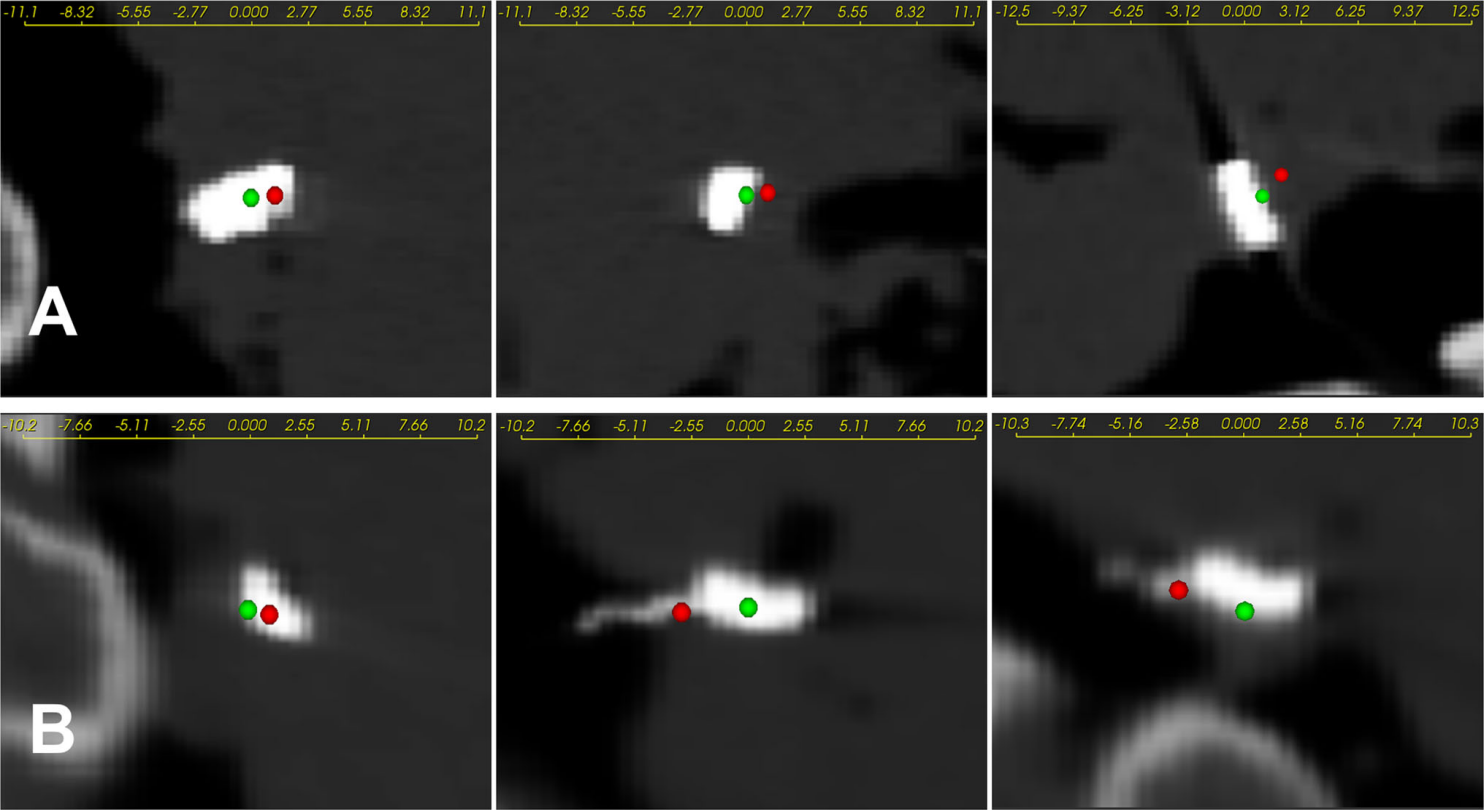
Postoperative CT images of the left (upper) and the right (down) intervention. The cross-sectional planes shown from left to right are sagittal, coronal and axial. Visualization of the actually reached target position as the center on the electrode’s surface closer to the target point (green sphere); and of the target point (red sphere)

**Fig. 7:**
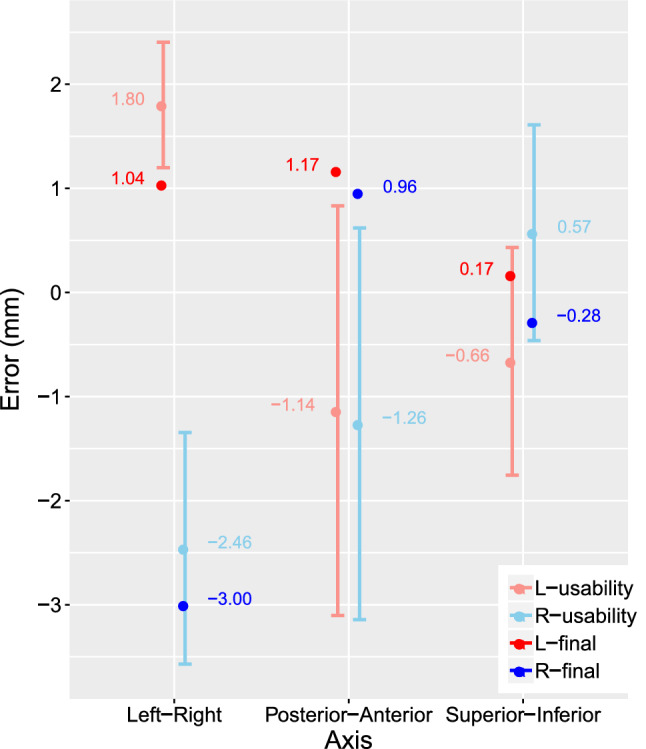
Localization errors (mean and standard deviation error bars) determined in the usability placement study (light colors) compared against final placement errors determined in the postoperative image (dark colors). The errors are shown along single axis per L-left and R-right side intervention

Table 3 gives the errors for final electrode positioning. Figure 6 visualizes the distance between actual and planned target positions. With one exception, in the right ear side intervention, the error was assessed for the position determined using the instrument after fixation of the implant (Video, Supp. 3, 08:16) as it was found that the electrode was displaced from the planned position (Fig. Supp. 5; Table, Supp. 6). Figure 7 shows a comparison between final placement errors against usability placement errors independently along each axis calculated in the CT coordinate system (Fig., Supp. 7).

**Table 3:** Experimentally determined final positioning errors

System	Side	Error [*x*, *y*, *z*]	Euclidean	Lateral	Depth
		(mm)	(mm)	(mm)	(mm)
VG	Left	[1.0, 1.2, 0.2]	1.58	1.18	1.04
VG	Right	$[-3.0, 1.0, -0.2]$	3.16	3.15	0.22

The [*x*, *y*, *z*] coordinates corresponding to the CT coordinate system (Fig., Supp. 7). “Lateral” and “Depth” corresponding to the local coordinate system of the electrode (Fig., Supp. 4)

## Discussion

### Clinical significance

An ABI involves complex neurosurgery to the critical areas of the brainstem and cumbersome anatomical localization of the CN. In the literature, the reported outcomes vary considerably between patients despite intraoperative assistance of EABRs [2]. Even slight electrode misplacements, among other hazards, contribute to poorer outcomes [6]. Enhancing spatial orientation might help reduce the risk of the latter and assist when anatomical landmarks are limited due to the surgical approach, surgeon’s experience [6] and, particularly, anatomical variations that arise from NF2 tumor removal [23].

In fact, in many other medical procedures, less invasiveness, accurate target localization, and reduced risk of complications were reported by physicians when using neuronavigation (or image-guided surgery) systems [26–28]. We hypothesize that these systems might offer solutions to surgeons confronting challenges during the conventional ABI approach, including more predictable and consistent surgical and audiological outcomes. However, a review of the literature reveals no prior use of these systems during ABI implantation.

We therefore demonstrated a microscope-integrated visually assisted framework that combines an intuitive and helpful guidance mode as well as highly accurate and automatized image registration. In particular, we used the same surgical instruments and electrode pads as in the clinical scenario. Nonetheless, they are not optimal for navigation precision, so certain design improvements could lead to still further improved results. However, it would subsequently require reiterating product and surgical tool development in compliance with medico-legal regulations without adding discomfort to surgeons.

### Key findings

The errors in final (1.58/3.16 mm) and usability ($2.32\pm 0.61$ mm) positioning appear to follow the same trend along single axes as shown in Fig. 7. It seems that both lateral and depth deviations meaningfully describe the error in their respective directions, as potentially both are susceptible to human errors given that the electrode is placed onto a curved anatomical surface.

There are no direct comparisons with our experimental results. In general, they correspond to the reported accuracy of intracranial electrode placement in stereoelectroencephalography where a mean target error of 2.89 mm (2.34 - 3.44 95% CI) was observed across several frameless stereotactic systems [27]. The intracranial and ABI electrodes are not directly comparable. The deep electrodes typically have a diameter $< 1$ mm and follow a predefined trajectory through the brain.

In line with our previous phantom study [10], the proposed visual interface achieves a lower localization error than cross-sectional views despite condensed information presented. However, slightly larger completion time and trajectory were observed. The surgeons, indeed, stated that guidance directly in the microscope was their preferred interface for the task at hand. They appreciated the possibility to be able to focus on the intervention itself while being guided toward the target with simple, intuitive visual cues that require minimum distractions and do not pose high cognitive load.

### Nasopharyngeal registration

Nasopharyngeal registration was central to establish reproducible and accurate spatial navigation in the vicinity of the CN. Our code enhances surgical workflow and reduces errors induced by human interactions. This is of special importance to maximize reproducibility due to the so-called silent loss of accuracy phenomena that occurs during the course of surgical procedures [29].

An early concept of this scheme was introduced for lateral skull base surgery and yielded submillimetric accuracy in a phantom setup [30]. We extended this scheme with a novel device [12]. Contrary to gold standard of invasive skull-implanted fiducial screws that need to be distributed around the head to reach optimal TRE, we aimed to bring fiducials in a minimally invasive way as closely as surgically possible to the target in order to achieve high registration and targeting accuracy at the surgical target.

We report a maximum (intrasurgical) dislocation of the spherical marker, $<1.72$ mm, which is in agreement with a recent endonasal magnetic tracker ($< 2$ mm) [28]. As reported in the clinical setting, this is better than a skin adhesive tracker ($< 3$ mm) [28]. The mean±standard deviation TRE measured on the implanted screw targets ($2.71 \pm 0.99$ mm) is higher compared to registration with screws ($<1.0$ mm [29]); approximately equal to adhesive marker registration ($2.49 \pm 0.86$ mm [31]); and lower than surface matching-based registration ($5.35 \pm 1.64$ mm [31]) as observed in clinical setup. However, higher TREs are to be expected in the current setting as the TRE grows proportionally to the distance of the target to the barycenter of the fiducial configuration [16]. The target points like the surgical point of interest were closer to the fiducial points, and thus TRE, as confirmed by numerical simulations (approx. 1.76 mm), can be expected to be lower.

### Electromagnetic tracking

Freehand navigation is advantageous in human-operated interventions, especially in the head-neck part, where the operating areas are small and cluttered [34]. One source of errors in our results is in part due to EMT. It yields inferior accuracy to optical tracking and is prone to ferromagnetic distortions present in operating theaters that could drastically degrade the accuracy [33]. Albeit, by prudently positioning the emitter, low-submillimetric accuracy can be observed in a laboratory [34] and clinical environment [33].

### Generalizability

This study has limitations. First, even though the implant has a plate geometry, only a single target point at the tool tip was covered during navigation in the visual display. We attempted to compensate for these by additional positional quality assurance checks on the implant surface after positioning. Second, since it is difficult to measure targets in the brain accurately, we rely on theoretical approximations of the TRE [16, 17]. There is a fair agreement between the measured and the predicted TRE within the error bounds (mean ± standard deviation) on the screws. The difference between the two TREs could be attributable to incorrectly estimated FLE. Our analysis shows that potentially the FLE is overestimated. The FLE can be calculated by [35]: $<{\mathrm{FRE}}^2>=(1-2/N)<FLE^2>$, where *N* is the number of fiducials and $<.>$ means “expected value of”. Our results are also limited with only two performed interventions and thus precluding detailed statistical analysis. The study does not compensate for the influence of cerebellar retraction and brainstem shift during surgery, which can exacerbate errors in registration and navigation. Further, the outer nose had to be cut laterally to the cartilaginous septum to allow placing the Rhinospider stent; conservation of the specimen changed the elasticity of the tissue so that the device could not be inserted into the nasopharyngeal space without risking damage. The embalmed specimen brain tissue can be very rigid, and surgical exposure of the CN without damage could be cumbersome.

### Future research

From this point, we believe that certain improvements would optimize surgical workflow. Any suitable atlas could be fused on the brainstem imaging to mark the nuclei more accurately, as this is routinely done for deep brain electrodes [36]. Furthermore, drawing from a recent study [37], by optimizing pre-operatively positioning of the entire dimensions of the electrode pad in the fused CT-MR images and predicting the categories of auditory performance or side effects resulting from non-auditory sensations before ABI activation. CT-MR combination might indeed be utilized for this task [2] as CT allows high-quality resolution of bony landmarks [37] while MR could provide better visibility of neural structures in the brainstem. Finally, bearing in mind that the electrode orientation contributes to auditory performance or side effects [37], estimating and presenting an intraoperative pose of the electrode array in the visual display could improve the positioning accuracy. However, this may be technically challenging with the current design of the electrode pad and forceps surgical instrument.

## Conclusion

We reported on the development and feasibility study of a novel visual guidance system that intraoperatively quantifies and facilitates ABI surgery. Our microscope setup lends itself into augmented reality visualization and is capable of informing the surgeon about the 3D distance with respect to the target without cluttering the surgeon’s view to the surgical scene. The nasopharyngeal registration technology shows promising performance for the potential application in neurosurgery.

## Supplementary Information

Below is the link to the electronic supplementary material.

Supplementary material 1 (mp4 443697 KB)

Supplementary material 2 (pdf 259 KB)
