# Supplementary material for: Novel microscope-based visual display and nasopharyngeal registration for auditory brainstem implantation: a feasibility study in an ex vivo model
Source: Int J Comput Assist Radiol Surg. 2021 Nov 18;17(2):261–70. doi: 10.1007/s11548-021-02514-x (PMC8784369; doi:10.1007/s11548-021-02514-x)

Supplementary Material

Article title: Novel microscope-based visual display and nasopharyngeal registration for auditory brainstem implantation: a feasibility study in an ex vivo model

Authors: M. Regodić, C. F. Freyschlag, J. Kerschbaumer, M. Galijašević, R. Hörmann and W. Freysinger

Fig, Supp 1

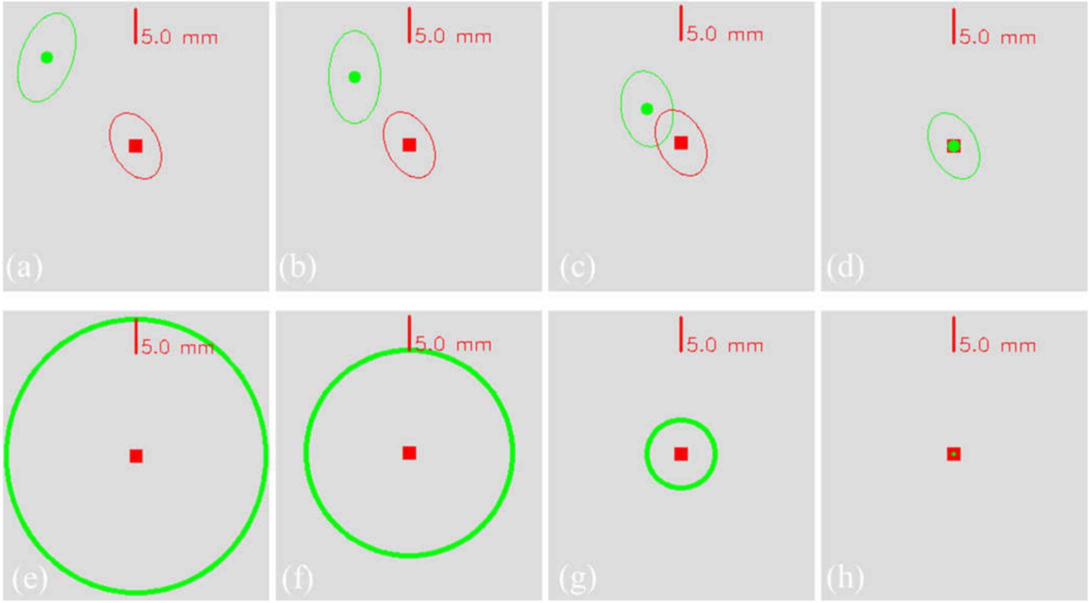

Green colored cues represent the tip position, whereas the red-colored cues the target. From left to right: illustration when the instrument tip is approaching the target visualized independently for lateral (A-D) and depth (E-H) distances. The vertical scale bar (red line) shows 5 mm for reference.

Fig, Supp 2

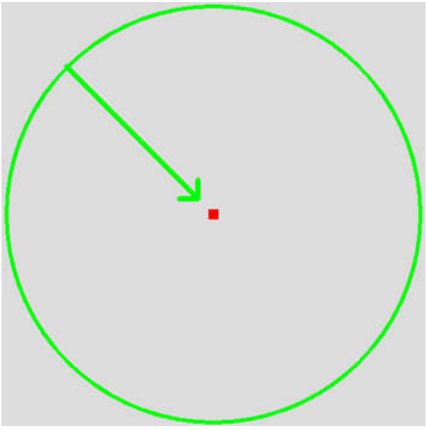

Visualization for an instrument-tip position classified as outside of the target range. Red Square – target; Green Arrow – direction from the tool tip to the target; Green Circle – depth displacement.

Video, Supp 3

A video clip that describes the experiment in an explanatory matter was attached as a supplemental digital content.

Figure, Supp 4

Left: A schematic example of the MED-EL's Mi1200 SYNCHRONY PIN ABI.

Right: The local coordinate system of the electrode in postoperative image. The origin is defined at the center of the surface of the imaged electrode closer to the target point in order to minimize the systematic error from the thickness of the electrode. X-Y form the lateral plane and Z the depth direction.

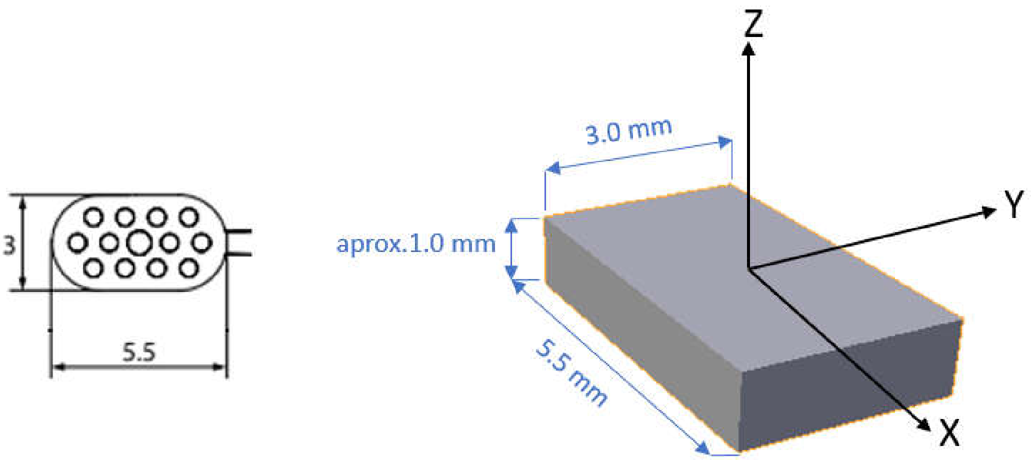

Figure, Supp 5

The postoperative image of the second intervention (right ear). Visualization of the actual position as a geometrical center of the electrode (green sphere) and the target position used during guidance. The latter was substituted with the position determined during QA-checks after final electrode positioning.

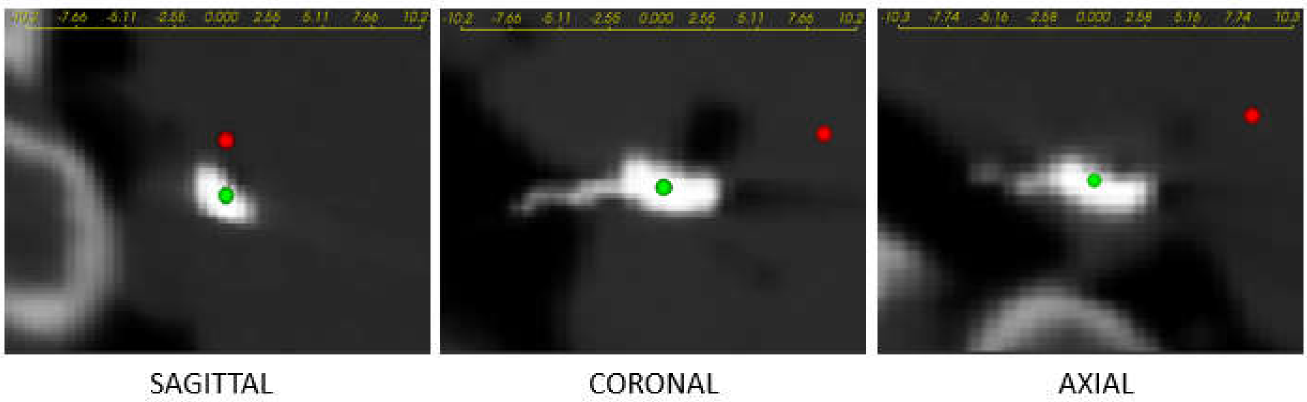

Table, Supp. 6

Determined errors for the second intervention for points as described in the figure above**.**

Intervention	Error	Euclidian distance
Right ear	3.36 x 8.28 x 2.82 mm^3	9.37 mm

Figure, Supp. 7

The CT coordinate system (left-posterior-superior; see https://itk.org/Wiki/Proposals:Orientation).

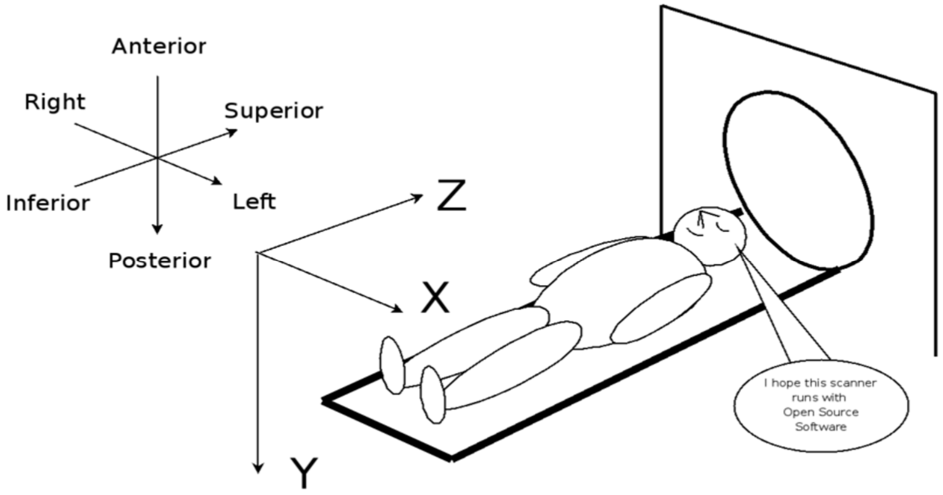

Supplement: Supplementary file 2 — Supplementary material 2 (pdf 259 KB) [file 11548_2021_2514_MOESM2_ESM.pdf]
